# Pre-hospital transthoracic echocardiography for early identification of non-ST-elevation myocardial infarction in patients with acute coronary syndrome

**DOI:** 10.1186/s13054-017-1929-1

**Published:** 2018-02-07

**Authors:** Ingo Bergmann, Benedikt Büttner, Elena Teut, Claudius Jacobshagen, José Hinz, Michael Quintel, Ashham Mansur, Markus Roessler

**Affiliations:** 1 Department of Anesthesiology, Emergency and Intensive Care Medicine, University Medical Center, University of Goettingen, Robert-Koch Str. 40, 37075 Göttingen, Germany; 2 Department of Cardiology and Pneumology, University Medical Center, University of Göttingen, Göttingen, Germany

**Keywords:** Pre-hospital, Transthoracic echocardiography, Emergency physician, Non-ST-elevation myocardial infarction, NSTEMI, Acute coronary syndrome, Regional wall motion abnormalities

## Abstract

**Background:**

Non-ST elevation myocardial infarction (NSTEMI) is a common manifestation of acute coronary syndrome (ACS), but delayed diagnosis can increase mortality. In this proof of principle study, the emergency physician performed transthoracic echocardiography (TTE) on scene to determine whether NSTEMI could be correctly diagnosed pre-hospitalization. This could expedite admission to the appropriate facility and reduce the delay until initiation of correct therapy.

**Methods:**

Pre-hospital TTE was performed on scene by the emergency physician in patients presenting with ACS but without ST-elevation in the initial 12-lead electrocardiography (ECG) (NSTE-ACS). A presumptive NSTEMI diagnosis was made if regional wall motion abnormalities (RWMA) were detected. These patients were admitted directly to a specialist cardiac facility. Patient characteristics and pre-admission and post-admission clinical, pre-hospital TTE data, and therapeutic measures were recorded.

**Results:**

Patients with NSTE-ACS (n = 53; 72.5 ± 13.4 years of age; 23 female) were studied. The 20 patients with pre-hospital RWMA and presumptive NSTEMI, and two without RWMA were conclusively diagnosed with NSTEMI in hospital. Percutaneous coronary intervention was performed in 50% of the patients presumed to have NSTEMI immediately after admission. The RWMA seen before hospital TTE corresponded with the in-hospital ECG findings and/or the supply regions of the occluded coronary vessels seen during PCI in 85% of the cases. The diagnostic sensitivity of pre-hospital TTE for NSTEMI was 90.9% with 100% specificity.

**Conclusions:**

Pre-hospital transthoracic echocardiography by the emergency physician can correctly diagnose NSTEMI in more than 90% of cases. This can expedite the initiation of appropriate therapy and could thereby conceivably reduce morbidity and mortality.

**Trial registration:**

Deutsche Register klinischer Studien, DRKS00004919. Registered on 29 April 2013.

## Background

Chest pain is in general the leading symptom that initiates the diagnostic measures and therapeutic interventions in patients with acute coronary syndrome (ACS), which is caused by unstable angina pectoris, non-ST-segment elevation myocardial infarction (NSTEMI) or ST-segment elevation myocardial infarction (STEMI) [1]. The correct treatment of myocardial infarction requires early detection and prompt transport to a percutaneous coronary intervention (PCI) facility. Standard 12-lead echocardiography (ECG) can unequivocally detect only myocardial infarction with *de novo* left bundle branch block (LBBB) or ST-segment elevation, which account for approximately 35% of ACS cases [1, 2]. Other rather unspecific ECG changes like persistent or transient ST-segment depression or T-wave inversion can only be suggestive of NSTEMI. Patients with ACS without ST-segment elevation (NSTE-ACS) are routinely treated in a general emergency facility, and the diagnosis of NSTEMI is often delayed [3]. The extent of the ischemic area may be the same [4], but the long-term prognosis of NSTEMI is worse than that of STEMI [5]. Since patients with chest pain but without evidence of ST-segment elevation or *de novo* LBBB present such a heterogeneous group in terms of the etiology of the pain (e.g. pulmonary embolism, aortic dissection, pericardial effusions, etc.), a risk assessment and definitive diagnosis is required before initiating any more invasive therapy [6]. Monitoring plasma levels of high-sensitivity (hs)-troponin as recommended in the 2011 [7] and 2015 [6] European Society of Cardiology (ESC) guidelines has reduced the time to confirmation of NSTEMI to 1 hour [8]. But high-risk or hemodynamically unstable NSTEMI patients would profit from an even more expedited diagnosis and consequent PCI, since this reduces mortality [3, 9]. In the 2011 ESC guidelines [7] the echocardiographic examination of all patients with ACS has already been recommended to detect regional wall motion abnormalities (RWMA) or identify differential diagnosis [10]. According to the fact that 12-lead ECG cannot confirm the diagnosis of NSTEMI, using pre-hospital TTE can help to detect NSTEMI on scene and thereby accelerate transportation of the patient to a primary PCI center.

In this proof of principal study we investigated whether pre-hospital TTE performed by an emergency physician could provide an even earlier diagnosis of NSTEMI in patients with NSTE-ACS. This could conceivably reduce the time to PCI, since the patients could be admitted directly to an interventional cardiology facility. An additional benefit of pre-hospital TTE would be the early differentiation between NSTEMI and other serious conditions, such as pulmonary embolism, aortic dissection, or cardiac tamponade that would shorten the time to the initiation of goal-oriented therapy.

## Methods

This study was approved by the ethics committee of the University of Göttingen Medical School (number 9/2/13) and was registered with the German registry of clinical studies under the clinical trial number DRKS00004919. It was conducted in association with the Emergency Medical Services (EMS) of Göttingen and the German Air Rescue Center at the University of Göttingen Medical School.

The inclusion criteria were age between 18 and 100 years and an initial diagnosis of ACS made by the emergency physician in the pre-hospital setting from the patient’s history and the clinical signs and symptoms. Exclusion criteria were previous myocardial infarction or New York Heart Association (NYHA) classification III or higher. Informed consent for further evaluation and data analysis was obtained on admission to the hospital. The emergency physicians (IB and MR) involved in the study were consultants for anesthesiology and intensive care medicine certified for focused cardiac sonography by the German Society of Anesthesia and Intensive Care Medicine (DGAI) and with long-term experience in emergency medicine.

At arrival on scene, a venous cannula was inserted, monitoring with 12-lead ECG, blood pressure and oxygen saturation was established, the patient was examined clinically and baseline values were recorded. The localization and severity of the thoracic pain on a standard numeric rating scale (NRS, 0 = no pain to 10 = worst pain imaginable) were documented and a 12-lead ECG printout was obtained. The latter was analyzed and a presumptive diagnosis of NSTE-ACS was considered if no ST-segment elevation or *de novo* LBBB was detected. Non-specific ECG changes (ST-segment depression, negative T-waves, *de novo* right bundle branch block) were consistent with the NSTEMI diagnosis.

Pre-hospital TTE was performed with a portable device (SonoSite®, Turbo) as focused cardiac ultrasound examination following a standardized 5-minute protocol focused on the emergency cardiac situation. The following 8 standard views were evaluated: apical four-chamber (1), five-chamber (2), two-chamber (3), three-chamber (4), parasternal long (5) and short (6) axes, and subcostal long (7) and short (8) axes. Left ventricular function was assessed and signs of other possible causes, such as aortic or mitral valve disorder, aortic dissection, pulmonary embolism, cardiac tamponade or hypovolemia, were sought. Views 1 to 4 were scanned for signs of RWMA. If present, they were assessed relative to cardiac geometry and classified as hypokinesia or akinesia. A diagnosis of NSTEMI was based on the combination of ACS symptoms, lack of ST-segment elevation, and RWMA. Myocardial infarction was excluded in the absence of the latter. The patients were then treated according to the ESC 2011 guidelines for NSTE-ACS [7] with sublingual glyceryl trinitrate for angina pectoris, acetylsalicylic acid, heparin, and morphine for pain according to NRS >3, if required. Hypoxemia was treated with titrated oxygen or non-invasive ventilation in patients with acute respiratory insufficiency.

The emergency physician then accompanied the patients to a hospital with specialized cardiology facilities where they were seen by the on-duty cardiologist. The baseline 12-lead ECG, the pre-hospital TTE findings and the presumptive diagnosis were communicated. If requested, the TTE loops recorded pre-hospitalization were demonstrated. The results of the PCI, if performed, were documented.

The definitive diagnosis of NSTEMI (NSTEMI group) or the exclusion of myocardial infarction (NoMi group) was based on the blood levels of cardiac biomarkers (e.g. hs-troponin) and data from cardiac catheterization, when performed. In patients with confirmed NSTEMI, the supply area of the affected coronary arteries seen in PCI was correlated with the localization of the RWMA observed pre-hospital to determine whether they corresponded. A study nurse blinded to the initial group allocation of the patients correlated the pre-hospital and in-hospital data for ejection fraction, evidence of right ventricular strain or valvular disorders, the occurrence of congestive heart failure or arrhythmias, or the necessity of, for example, cardiopulmonary resuscitation, cardioversion, catecholamine therapy, or mechanical ventilation. The 30-day and 90-day survival rates were recorded. All clinical findings and documented clinical records were independently checked for plausibility and accuracy by two study doctors (BB and AM), before being discussed by the whole study team at regular intervals.

### Statistical analysis

The primary outcome factors were sensitivity and specificity of RWMA detected by pre-hospital TTE in diagnosing or excluding NSTEMI. Secondary outcomes were in-hospital treatment, complications, and mortality.

The data were analyzed with the statistics program StatSoft® (Dell Inc., Texas, USA). Continuous data were tested for normal distribution with the Kolmogorov-Smirnov test. Normally distributed data were described with mean and standard deviation and compared with Student’s *t* test. Non-parametric and ordinal data were described with median and range and compared by the Mann–Whitney U test. Categorical data were analyzed as percentages and compared using Fisher's exact test. A *p* value <0.05 was defined as statistically significant.

## Results

Fifty-three patients presenting with symptoms of acute coronary syndrome without ST-segment elevation were enrolled in the study in the period August 2013 to January 2015. All focused echocardiography findings were identified by the emergency physician on scene; there was no re-reading of the images by an expert afterwards. NSTEMI was confirmed in 22 patients, while myocardial infarction was excluded as the cause of symptoms in 31 (NoMi, no myocardial infarction). The emergency physician who conducted the focused cardiac ultrasound examination had knowledge of the ECG findings and carried out the pre-hospital TTE regardless of these findings. This complies with the ESC 2015 guidelines, suggesting that unspecific ECG changes should be treated like normal ECG findings, whereas, well-conducted ECG confirms the diagnosis of myocardial infarction. The two groups did not differ with regard to anthropometric data (Table 1). The median severity of the angina pectoris pain, as measured according to the standard NRS for clinical pain measurement, was moderate and identical in both groups (NSTEMI 3.0 (range 0–5); NoMi 3.0 (0–5); *p* = 0.54).

**Table 1:** Patient characteristics (mean ± SD)

	NSTEMI, n = 22	NoMI, n = 31	*p*
Age (years)	75.1 ± 12.4	70.1 ± 14.0	0.24
Height (cm)	170.4 ± 7.5	169.6 ± 14.2	0.81
Weight (kg)	80.0 ± 15.1	86.7 ± 18.5	0.17
Male/female (*n*)	15/7	15/16	0.15
Smoking (%)	45.0	43.3	0.91
Diabetes mellitus (%)	47.6	23.3	0.07

*NSTEMI* non-ST elevation myocardial infarction, *NoMI* no myocardial infarction

Blood pressure tended to be lower in patients with NSTEMI. Sinus rhythm in the initial ECG recording was equally frequent in both groups; 32% of patients in the NSTEMI group exhibited normal ECG and 68% of patients in the NSTEMI group had unspecific ECG changes. In the NoMi-group 61% had normal ECG, but 39% had unspecific ECG changes (Table 2).

**Table 2:** Pre-hospital clinical findings (median [range])

	NSTEMI, *n* = 22	NoMI, *n* = 31	*p*
Heart rate (min^−1^)	76.0 (35–190)	83.0 (60–150)	0.38
SAP (mmHg)	141.5 (70–260)	150.0 (80–230)	0.07
DAP diast (mmHg)	80.0 (50–120)	90.0 (60–170)	0.03
ECG findings			
Sinus rhythm in first ECG (*n* (%))	18 (81.8)	25 (80.7)	0.91
ST-segment depression (*n* (%))	16 (72.7)	7 (22.6)	<0.001
Anterior leads (*n* (%))	11 (50.0)	6 (19.4)	0.02
Posterior leads (*n* (%))	3 (22.7)	2 (6.5)	0.08
*De novo* negative T-waves (*n* (%))	2 (9.1)	2 (6.5)	0.72
*De novo* RBBB (*n* (%))	4 (18.2)	6 (19.4)	0.49

*ECG* electrocardiogram, *DAP* diastolic arterial pressure, *NoMI* no myocardial infarction, *RBBB* right bundle branch block, *SAP* systolic arterial pressure

Myocardial RWMA were seen in 20 NSTE-ACS patients with ACS, who were therefore classified as NSTEMI. The most frequent RWMA localizations were the posterior and lateral walls (Table 3). The sites of the RWMA seen in the pre-hospital TTE coincided in 85% of the cases with those found in the subsequent examinations in the hospital in the course of PCI or repeat in-hospital ECG (Table 3). The ejection fraction was re-determined in the hospital in 31 of the 53 patients with good correlation between the two measurements (Table 3).

**Table 3:** Echocardiography findings and in-hospital interventions, NSTEMI/NoMI, median (range) (mean, SD)

Final diagnosis	NSTEMI, *n* = 22	NoMI, *n* = 31	*p*
Pre-hospital TTE			
Patients with/without RWMA (*n*)	20/2	0/31	
Location of RWMA (multiple selections possible) (calculations refer to patients with pre-hospital diagnosis)			
RWMA septal (*n* (%))	1 (5)	0	-
RWMA lateral (*n* (%))	10 (50)	0	-
RWMA anterior (*n* (%))	5 (25)	0	-
RWMA posterior (*n* (%))	14 (70)	0	-
EF (%) pre-hospital	38.0 ± 11.7	52.0 ± 7.2	<0.001
In-hospital echocardiography			
EF (%) (calculations refer to patients with final diagnosis)	35.3 ± 20.3 (in 16 of 22)	49.9 ± 17.3 (in 15 of 31)	0.06
Immediate PCI (*n* (%))	10 (50%)	4 (12.9)	0.004
Sensitivity for correct diagnosis	90.9%	-	
Selectivity for correct diagnosis	100%	-	
Positive predictive value	100%	-	
Negative predictive value	94%	-	
Correlation between pre-hospital observed RWMA and perfusion area of affected coronary arteries determined in hospital	85.0%	-	

Ejection fraction (EF) <40% indicates poor outcome

*NSTEMI* non-ST elevation myocardial infarction, *NoMI* no myocardial infarction, *PCI* percutanous coronary intervention, *RWMA* regional wall motion abnormalities, *TTE* transthoracic echocardiography

NSTEMI was conclusively diagnosed in the 20 patients with RWMA and in an additional 2 patients without. No evidence of myocardial infarction was found in any patient with NSTE-ACS with normal wall motion in the pre-hospital TTE. RWMA in pre-hospital TTE had 100% specificity and 100% positive predictive value for predicting myocardial infarction in our patients. However, normal wall motion was false negative for NSTEMI in 9.1% and had a negative predictive value of 94%.

The patients diagnosed with NSTEMI had a more complicated course with a greater need for supportive measures (Table 4), a longer duration of stay in the hospital, and greater mortality than patients with NoMi (Table 5).

**Table 4:** Critical pre-hospital events and necessary therapeutic measures in patients with the corresponding final diagnoses

	NSTEMI, *n* = 22	NoMI, *n* = 31	*p*
Arrhythmias (*n* (%))	3 (13.6)	6 (19.4)	0.58
CPR (*n* (%))	4 (18.2)	2 (6.5)	0.17
Requiring circulatory support (e.g. catecholamines (*n* (%)))	9 (40.9)	3 (9.7)	0.017
NIV (*n* (%))	12 (54.6)	2 (6.5)	<0.001

*CPR* cardiopulmonary resuscitation, *NSTEMI* non-ST elevation myocardial infarction, *NoMI* no myocardial infarction, *NIV* non-invasive ventilation

**Table 5:** Duration of hospital stay and cumulative mortality

	NSTEMI, *n* = 22	NoMI, *n* = 31	*p*
Duration of hospital stay (median (range))			
Stay in ICU (days)	5 (1–27)	2 (0–28)	0.03
Total in hospital (days)	10.5 (1–27)	3.0 (1–28)	0.02
Cumulative mortality			
In-hospital (%)	18.2	3.2	0.09
90-Day (*n* (%))	7 (31.8)	1 (3.2)	0.006

*ICU* intensive care unit, *NSTEMI* non-ST elevation myocardial infarction, *NoMI* no myocardial infarction

## Discussion

This is the first report of pre-hospital focused transthoracic ECG on scene by an anesthesia-specialist emergency physician in the diagnostic workup of patients with acute coronary syndrome (ACS) to detect those with NSTEMI. Patients with ACS with RWMA in the initial pre-hospital TTE were given a presumptive NSTEMI diagnosis, and this was compared to the definitive in-hospital diagnosis based on laboratory values and cardiac-catheter examination. We believe that this is an important pre-clinical investigation since an early diagnosis can shorten the crucial time to the initiation of correct therapy in patients with NSTEMI. The patients with a presumptive diagnosis of NSTEMI were admitted primarily to a cardiology department with facilities for invasive therapy, and 50% were instrumented immediately after hospital admission. This illustrates the importance of an early differentiation of patients with NSTE-ACS.

The percentage of patients with NSTE-ACS and NSTEMI in our collective was 42%, which is midrange in the published rates of 14–61% [4, 11]. The comparison of the pre-hospital TTE findings and the in-hospital confirmed diagnosis demonstrated that the pre-hospital TTE assessment of patients with NSTE-ACS correctly diagnosed NSTEMI with 90.9% sensitivity and 100% specificity. The corresponding positive and negative prediction values were 100% and 94%. In-hospital catheter data and/or repeat ECG confirmed the pre-hospital TTE assessment of the involved coronary arteries in 85% of the cases. Previous studies have shown that immediate TTE examination on the patient’s arrival in the emergency department allows early detection of the RWMA that indicate myocardial ischemia [4, 12]. Based on these findings the 2015 ESC guidelines [6] recommend that TTE should be performed on every patient with NSTE-ACS requiring emergency care in order to minimize the delay between hospital admission and initiation of PCI in patients with NSTEMI.

The patients in our study with confirmed NSTEMI had a 90-day mortality rate of 35%, which is considerably higher than the otherwise reported rate of 5–15% for patients with NSTEMI [5, 13, 14]. This is possibly due to the fact that our patients were more seriously ill. The initial mean ejection fraction in the NSTEMI group was 38.0% (±11.7), and 15 patients (68%) had evidence of acute heart failure. Six patients (27%) had an initial oxygen saturation of less than 90%, and 12 (55%) required non-invasive ventilation during transport to hospital. Four patients (18%) required pre-hospital cardiopulmonary resuscitation. The reason for this preponderance of more compromised patients in our collective is probably because the dispatcher only sends the physician-manned emergency vehicles to patients with life-threatening complaints. But these would be the ones most likely to profit from early diagnosis using pre-hospital TTE and subsequent targeted therapy. Furthermore, ECG is recommended in the differential diagnostic workup of patients with cardiovascular compromise, particularly in hemodynamically unstable patients with ACS [15].

The pre-hospital detection of patients with NSTEMI allowed us to present them directly to specialized cardiac facilities. Pre-hospital TTE also allowed us to monitor the effect of our therapeutic measures (e.g. vasoactive and inotropic drugs). TTE might not be available to all emergency services, but Sobczyk et al. [16] showed that correct patient allocation was made possible by a 5-minute focused cardiac ultrasound examination, which could be performed by trained non-cardiologist emergency room physicians [16]. This measure should be adopted by all emergency departments that have access to a transthoracic ultrasound device in order to comply with the 2015 ESC guidelines [6, 15].

Some limitations of our study must be discussed. Pre-hospital TTE was conducted by two emergency physicians who were very familiar and highly experienced in conducting focused cardiac ultrasound in the pre-hospital setting. This means that other centers without an emergency physician with extensive ECG experience may not achieve the same results. A condition for generalizability is the necessity to teach the involved emergency physician and give them the chance to train in frequent, focused, cardiac ultrasound examinations for many years before expecting from them to provide reliable, focused, cardiac ultrasound examinations in the pre-hospital setting. In addition, in our study we have excluded patients with preexisting myocardial infarction and those with NYHA III or higher. This fact might have contributed significantly to the results of the study. Furthermore, focused cardiac ultrasound without measuring wall thickness, as conducted in our study, may not be useful in patients with non-ST elevation myocardial infarction, who have had prior myocardial infarction or severe heart disease with chronic wall motion abnormalities, in order to distinguish between new and old wall motion abnormalities.

In conclusion, the results of the present study suggest that focused ECG in patients with suspicion of non-ST elevation myocardial infarction, performed by emergency physicians to assess regional wall motion abnormalities, may be useful in confirming the diagnosis of NSTEMI and thereby accelerate the transportation of the patient to a primary hospital with a PCI center.

## Conclusions

Focused, cardiac ultrasound examination shortens the crucial time until patients with NSTEMI receive proper and necessary treatment. This is best performed before hospital admission but should otherwise be performed immediately on admission in all patients with non-ST elevation acute coronary syndrome.

## Abbreviations

ACS: Acute coronary syndrome
DGAI: German Society of Anesthesia and Intensive Care Medicine
ECG: Echocardiography
EMS: Emergency Medical Services
ESC: European Society of Cardiology
LBBB: left bundle branch block
NRS: numeric rating scale
NSTE-ACS: ACS without ST-elevation
NSTEMI: Non-ST elevation myocardial infarction
NYHA: New York Heart Association
PCI: percutaneous coronary intervention
RWMA: regional wall motion abnormalities
STEMI: ST elevation myocardial infarction
TTE: Transthoracic echocardiography
